# Substantial viral diversity in bats and rodents from East Africa: insights into evolution, recombination, and cocirculation

**DOI:** 10.1186/s40168-024-01782-4

**Published:** 2024-04-10

**Authors:** Daxi Wang, Xinglou Yang, Zirui Ren, Ben Hu, Hailong Zhao, Kaixin Yang, Peibo Shi, Zhipeng Zhang, Qikai Feng, Carol Vannesa Nawenja, Vincent Obanda, Kityo Robert, Betty Nalikka, Cecilia Njeri Waruhiu, Griphin Ochieng Ochola, Samson Omondi Onyuok, Harold Ochieng, Bei Li, Yan Zhu, Haorui Si, Jiefang Yin, Karsten Kristiansen, Xin Jin, Xun Xu, Minfeng Xiao, Bernard Agwanda, Sheila Ommeh, Junhua Li, Zheng-Li Shi

**Affiliations:** 1 BGI Research, Shenzhen, 518083 China; 2 CAS Key Laboratory of Special Pathogens and Biosafety, Wuhan Institute of Virology, Chinese Academy of Sciences, Wuhan, China; 3 Mammalogy Section, National Museums of Kenya, Nairobi, Kenya; 4 Veterinary Services Department, Kenya Wildlife Service, Nairobi, Kenya; 5 Center for Animal Science, Queensland Alliance for Agriculture & Food Innovation, The University of Queensland, St Lucia, QLD 4072 Australia; 6 Department of Zoology, Entomology and Fisheries Sciences, School of BioSciences, Makerere University, Kampala, Uganda; 7 Laboratory of Genomics and Molecular Biomedicine, Department of Biology, University of Copenhagen, Copenhagen, Denmark; 8 University of Chinese Academy of Sciences, Beijing, China; 9 College of Life Sciences, University of Chinese Academy of Sciences, Beijing, 100049 China; 10 Shenzhen Key Laboratory of Unknown Pathogen Identification, BGI Research, Shenzhen, 518083 China; 11 Sino-Africa Joint Research Center, Chinese Academy of Sciences, Wuhan, China; 12 Hubei Jiangxia Lab, Wuhan, 430071 China

**Keywords:** Virome, Evolution, Viral surveillance, Metatranscriptome

## Abstract

**Background:**

Zoonotic viruses cause substantial public health and socioeconomic problems worldwide. Understanding how viruses evolve and spread within and among wildlife species is a critical step when aiming for proactive identification of viral threats to prevent future pandemics. Despite the many proposed factors influencing viral diversity, the genomic diversity and structure of viral communities in East Africa are largely unknown.

**Results:**

Using 38.3 Tb of metatranscriptomic data obtained via ultradeep sequencing, we screened vertebrate-associated viromes from 844 bats and 250 rodents from Kenya and Uganda collected from the wild. The 251 vertebrate-associated viral genomes of bats (212) and rodents (39) revealed the vast diversity, host-related variability, and high geographic specificity of viruses in East Africa. Among the surveyed viral families, *Coronaviridae* and *Circoviridae* showed low host specificity, high conservation of replication-associated proteins, high divergence among viral entry proteins, and frequent recombination. Despite major dispersal limitations, recurrent mutations, cocirculation, and occasional gene flow contribute to the high local diversity of viral genomes.

**Conclusions:**

The present study not only shows the landscape of bat and rodent viromes in this zoonotic hotspot but also reveals genomic signatures driven by the evolution and dispersal of the viral community, laying solid groundwork for future proactive surveillance of emerging zoonotic pathogens in wildlife.

Video Abstract

**Supplementary Information:**

The online version contains supplementary material available at 10.1186/s40168-024-01782-4.

## Background

Viruses infect a wide range of wildlife species. Among the virus carriers, bats (Chiroptera) and rodents (Rodentia) have received the most attention due to their unique immune systems and natural history features and the role of viral reservoirs [1–3]. Although most viruses are host-specific, rapid viral evolution and increasing contact between wildlife and humans or domestic animals have enabled the emergence of many zoonotic pathogens, posing a major risk to public health worldwide. Understanding how viruses evolve and transmit within and among wildlife species is critical for efficient and proactive pathogen surveillance.

During viral evolution, rapid mutation and frequent recombination contribute to substantial genomic diversity and genetic plasticity to facilitate host adaptation. In particular, recombination plays a pivotal role in developing virus‒host compatibility during the emergence of SARS, SARS-CoV-2, and many other zoonotic pathogens [4–6], as it effectively purges deleterious mutations and accumulates beneficial mutations through the exchange of genetic components between viruses infecting the same host [7].

Given the lack of sufficient knowledge of the genomic diversity and population structures of natural viral communities, fully understanding the mechanisms underlying viral evolution in wildlife is still difficult. Different viral groups exhibit substantial differences in replication fidelity [8], genome replication [9], viral abundance [10], and recombination frequency [11], leading to distinct intra-host and inter-host population dynamics. In addition, host migration and geographic ecology play major roles in viral transmission across geographical regions and in adjusting the local diversity of viral communities [12]. Consequently, the complex interactions among viral biological features, host traits, and stochastic processes pose great challenges when predicting the distribution and genomic diversity of wildlife viral communities.

Currently, most viral surveillance methods focus on a few taxa groups by targeting conserved regions of viral genomes. Despite the advantages of cost-effectiveness and procedural simplicity, these detection methods can lead to bias. The preselection of genomic regions not only limits information on viral genomic evolution but also frequently prevents the surveillance of other viral groups (e.g., astroviruses and picornaviruses) with zoonotic potential [13]. Additionally, variation in viral detection methods limits the integration of data among surveillance programs, complicating comparisons across geography or host species. Despite considerable findings from genomic comparisons of specific zoonotic viruses, limited research has explored the genomic diversity and genetic exchange of the whole viral community in wildlife. This may provide insights into geographic dispersal and spillover-related features in neglected viral groups.

Because of its ability to characterize the entire virome, metatranscriptomic sequencing (sequencing of all the molecules from total RNA) has been widely used to unveil viral diversity worldwide [14–17]. With the decrease in sequencing cost, metatranscriptomic sequencing at the sample level (28 viruses from 161 animals) [18] has been shown to be more effective for characterizing vertebrate-associated viruses than pooled libraries (102 viruses from 239 pools of 1941 animals) [19]. Consequently, ultradeep metatranscriptomic sequencing is expected to offer significantly higher resolution, enabling systematic comparisons of evolutionary and population patterns across diverse viral groups.

Among the areas under investigation, Africa is a high priority for surveillance due to its rich mammalian diversity and the emergence of many zoonotic pathogens [20]. To explore the virome diversity, evolutionary features, and viral circulation in this zoonotic hotspot, we performed ultradeep metatranscriptomic sequencing of 1282 samples collected from 844 bats and 250 rodents from East Africa, providing valuable genomic resources for viral surveillance in the future. We revealed substantial local viral diversity related to viral sharing, cocirculation, recombination, and geographic transmission in multiple viral groups, demonstrating the value of deep sequencing to trace genomic interactions and target viral hotspots for effective pathogen surveillance in wildlife.

## Methods

### Sample collection

Bat and rodent samples were collected from 49 locations in Kenya and Uganda between 2014 and 2019 (Additional file 1: Table S1; Additional file 2: Fig. S1). For bats, the samples were collected from roosts in caves, trees, and inhabited and abandoned buildings. Clean polythene sheets (2.0 × 2.0 m) were spread at known bat roosting sites from the first evening (18:00) to the next morning (6:00) for the collection of fresh fecal pellets. The fecal pellets were then collected and placed in RNAlater Stabilization Solution (QIAGEN, Germany). For rodents, tissue samples comprising lung, kidney, and liver tissues were collected. All the samples were stored at − 80 °C. This study was approved by the Research and Ethics and Committee of the Kenya Wildlife Service (KWS) under permit KWS/BRM/5001, the Uganda National Council for Science and Technology (UNCST) under permit NS644, and the Uganda Wildlife Authority under permit ID UWA/COD/96/05.

### Clustering of geographic locations

The 49 sampling locations were clustered into 16 geographic sites using the R package hclust (complete method) based on the geographic spherical distance. The performance of the cluster was evaluated based on the maximum spherical distance within a clustered site from 70 to 110 km by the Silhouette algorithm. The silhouette score ranges from − 1 to + 1, where a high value indicates that the location is well-matched to its own cluster and poorly matched to neighboring clusters. The top mean silhouette score (0.6) within the cluster is based on thresholds over 90 km.

### RNA library construction and sequencing

For the 1282 samples, RNA was extracted using QIAamp Viral RNA Mini Kit and QIAamp 96 Virus QIAcube HT Kit (QIAGEN, Hilden, Germany). To account for potential PCR-mediated recombination [21], nucleic acid extraction and library preparation were performed for each sample. The sequencing libraries were prepared using the MGIEasy RNA Library Prep Kit V3.0. Briefly, the RNA was fragmented, reverse-transcribed, and synthesized into double-stranded cDNA. The unique dual-indexed cDNA was circulated, and the rolling-circle replication was used to generate DNA nanoball (DNB)-based libraries. The constructed libraries were subsequently sequenced on the DNBSEQ T series platform (MGI, Shenzhen, China) to generate metatranscriptomic data of 150-bp paired-end reads.

### Clean-up of the raw reads

For each sample, most reads from ribosomal RNA (rRNA) were first removed using URMAP (version 1.0.1480) [22]. Adapters and low-quality reads were removed using fastp (version 0.20.1, -q 20 -n 2 -y -c -p -G) [23]. The reads with duplicates, low complexity, and remaining rRNA sequences were removed using SOAPnuke (version 2.1.5, -l 20 -q 0.2 -n 0.02 -4 50) [24], PRINSEQ +  + (version 1.2, -lc_entropy = 0.5 -lc_dust = 0.5) [25], and SortMeRNA (version 4.3.2) [26], respectively (Additional file 2: Fig. S2). All software was run with the default settings unless otherwise specified.

### Host species identification

For each sample, de novo assembled contigs were compared against a customized database of three mitochondrial markers (*cytB*, *cox*1, and *nad*1 sequences from GenBank, see Additional file 3) using BLAT (version 35) [27]. For each marker, the species of the best-matched mitochondrial sequences with less than 5% nucleotide difference were used to annotate the species of the marker. The host species of the sample were then defined using the majority voting principle based on the annotation of the three markers. The phylogenies of the identified host species (Additional file 2: Fig. S1) were derived from public subsets of the mammalian phylogeny (http://vertlife.org/phylosubsets) [28].

### Viral sequence identification

The overall workflow for viral sequence identification is illustrated in Additional file 2: Fig. S2. The filtered reads were de novo assembled using MEGAHIT (version 1.2.9) [29] with default settings. Contigs with at least 1000 bp were compared against viral proteins from the nonredundant (NR) protein database and the RefSeq and IMG-VR databases using the BLASTX alignment mode (*e* value < 10^–5^) of DIAMOND (version 0.9.36.137) [30]. To remove nonviral sequences, the matched contigs were assessed using the Contig Annotation Tool (CAT, version 5.2) with the entire NR database (available as of Nov 27, 2020) [31]. Contigs classified under the kingdom ‘‘Viruses’’ were further compared against the nucleotide (NT, available as of Nov 27, 2020) database using BLASTN (version 2.11.0 +). Contigs best matched to non-viral sequences in the NT database were removed. The putative viral contigs were also compared against a customed database comprising genomes of the order Chiroptera and Rodentia (Additional file 4) using BLAT. Chimeric regions flanking the viral contigs were cropped using a customed python script. The viral sequences shorter than 1000 nucleotides were removed. The proteins translated from each viral contig were compared against representative replication-associated proteins obtained from the NR database using the BLASTP alignment mode (*e* value < 10^–5^) in DIAMOND (version 0.9.36.137) [30]. The aligned contigs were defined as putative viruses. For RNA viruses, only the viruses that were matched to manual curated conserved domains of RNA-dependent RNA polymerase (RdRp) from the conserved domain database [32] (Additional file 5) with *e* value < 10^–5^ using either Position-Specific Iterated BLAST (PSI-BLAST, version 2.11.0 +) [33] or Reverse Position-Specific BLAST (RPS-BLAST, version 2.11.0 +) were considered further. The family of each viral sequence was annotated according to the taxonomy of known viral sequences from the NR database with the highest similarity. To annotate vertebrate-associated viruses, all viral sequences were compared against proteins in the Virus‒Host Database (http://www.genome.jp/virushostdb/, release 209) using the BLASTX alignment mode (*e* value < 10^–5^) in DIAMOND (version 0.9.36.137) [30]. Only the viral sequences that were most similar to a vertebrate-associated virus (with the host under the clade “Vertebrata”) were selected for further analyses. The completeness of each viral sequence was assessed using CheckV (version 0.8.1) [34]. The viral sequences were then clustered using CD-HIT-EST (version 4.8.1) [35] at 80% (vANI80) and 95% average nucleotide identity (vANI95) with additional parameter -g 1 -d 0 -aS 0.80. Within each clustering level, the viral sequence with the longest length was selected to represent the viral vANI80/vANI95 cluster. For each cluster, a representative genome with a completeness of less than 50% was subjected to genome quality improvement. Based on the vANI95 clustering, we used MEGAHIT (version 1.2.9) [29] with default settings to re-assemble pooled reads across samples containing sequences of the same vANI95 cluster. After re-assembly, BLASTN was employed to align the newly assembled sequences against their corresponding lower-completeness genomes. We retained a result if the newly assembled genome was at least 20% longer than the previous one. Further, viral contigs from the same sample were scaffolded under the guidance of a closely related reference genome when applicable. Briefly, viral contigs were compared against the assembled representative genomes and NT database using BLASTN. For each representative genome, the most closely related complete genome (with at least 70% sequence identity) was selected. The contigs were ordered and connected linearly based on their alignment to the reference, with gaps filled with a series of ‘Ns’ to denote unknown sequences between contigs. All software were run with the default settings unless otherwise specified.

### Phylogenetic analysis

For each viral family, the replication-associated proteins of each vANI95 were aligned with representative marker proteins of the same family using the E-INS-i algorithm of MAFFT (version 7.475) [36] and trimmed using TrimAI (version 1.4) [37]. Sequences with fewer than 50 amino acids within the alignment were removed. Maximum likelihood trees were constructed using IQ-TREE multicore (version 2.1.2) [38] with 1000 bootstrap replicates (default settings).

### Recombination analysis

Among the vANI95 representatives, the representative genome of each vANI95 (query) was compared to the other assembled representative genomes and the coronavirus genomes from GenBank within each sliding window of 1000 bp shifted by 500 bp each step across the representative genome using BLASTN. To identify potential recombination source, the matched genomes with the highest bitscore and at least 60% identity in each sliding window were selected and aligned with the query genome using MAFFT (version 7.475) with default settings [36]. Recombination tests were performed with RDP, GENECONV, MaxChi, BootScan, SiScan, and 3Seq using RDP4 with default settings (window size > 300 bp; step size > 30 bp) and reviewed manually using RECAN (version 0.1.2) [39]. Only putative recombinations (recombination segment > 300 bp) that passed at least four recombination tests were considered valid recombination events. Genomic similarity visualization was performed with a 600 bp window size and a 60 bp step size using Simplot (version 3.5.1) [40]. For each viral family, the viral genomes involved in the recombination events were connected and visualized using Circos (version 0.69–8) [41]. Within the vANI95 cluster, which has a sufficient sample size and genomic variability, viral lineages and regions of recent recombination within the alignment of consensus genomes were defined using fastGEAR [42] with default settings and visualized using the plotRecombinations script of fastGEAR [42].

### Comparison of evolutionary conservation among marker proteins

For each viral genome, potential open reading frames for protein translation were predicted using the getorf function of the EMBOSS software package (version 6.5.7.0) [43]. The replication-associated proteins (RAPs) and viral entry proteins (VEPs) were annotated based on manually curated HMM profiles for each viral family using hmmsearch in HMMER software (version 3.3.2) [44]; these proteins were subsequently trimmed using SeqKit (version 2.1.0) [45]. The HMM profiles of the RdRp core motif were derived from multiple sequence alignments of the RdRp database using RdRp-scan [46]. The HMM profiles for the remaining proteins were curated from the corresponding Pfam profiles. The detected marker proteins were aligned using the E-INS-i algorithm of MAFFT (version 7.475) [36]. The multiple sequence alignment was subjected to the calculation of amino acid identity (AAI) between every pair of vANI95 representatives from the same viral family. The network showing the VEP-AAI among viral pairs with a RAP-AAI > 90% was visualized using Cytoscape (version 3.10.0) [47].

### Quantification of viral abundance

The clean reads from each library were mapped to all the representative viral genomes using Bowtie2 with “very-sensitive-local” mode (version 2.4.2) [48]. Only the viral mapping records with at least 40% genome coverage and a 1000 bp coverage length were considered further. For each sample, the viral species with the longest coverage length and other species with a mismatch rate of less than 6% were retained. To allow intraspecific diversity within the same sample, the vANI95 representative with the longest coverage length, and other vANI95s from the same vANI80 clusters that had mismatch rates of less than 1%, was retained. For each vANI95 cluster, the abundance was calculated as the proportion of mapped reads across all the clean reads. For the comparison of viral composition, only viral records with a proportion of viral reads greater than 5*10^–7^ were retained.

### Phylogenetic diversity

The mean pairwise distance (MPD) and the mean nearest taxon distance (MNTD) statistics and their standardized effect sizes (SESs) were calculated for each host family (with at least three viral records) using the R package picante [49]. The MPD and MNTD are the mean distance between all pairs of viral sequences within a host family and the mean distance between the viral sequence and its nearest phylogenetic neighbor within a host family, respectively. The SES values of MPD and MNTD were used to estimate the difference in the phylogenetic distances between the observed communities and null communities by reshuffling tip labels 1000 times on the phylogeny. The MPD (and SES) between viral pairs from distinct host families were estimated using the function comdist of the R package phylocomr. The interhost MPDs were used to cluster host families into dendrograms according to their evolutionary similarity using the R function hclust.

### Variant detection in host mitochondrial genomes

For major bat species, the reference mitochondrial genome for read mapping was obtained from GenBank when available or from the mitochondrial genome assembled from the sequencing data. For each sample, clean read data were mapped to the reference mitochondrial genome using Bowtie2 (version 2.4.2) [48], which was subsequently subjected to single nucleotide variant (SNV) detection using BCFtools (version 1.14) [50] with default settings. Single nucleotide polymorphisms were removed if they occurred in less than 75% of samples, and samples with less than 50% of the SNPs from the previous step were removed.

### Variant detection

The clean reads of each sample were mapped to the representative sequence of each vANI95 using Bowtie2 (version 2.4.2) [48]. From the resulting BAM files, the consensus single nucleotide variants (cSNVs) between hosts were detected using Freebayes (https://github.com/freebayes/freebayes) under haploid mode (-p 1 –standard-filters). For each vANI95 cluster, the samples missing data from more than 20% of the cSNV sites were removed first, and the cSNV sites were removed when they were present in less than 75% of the samples. The retained cSNVs of each sample were converted into consensus sequences and aligned across all the corresponding samples. The intrahost single nucleotide variants (iSNVs) were detected using the variant caller LoFreq (version 2.1.5) with default filters and a cutoff of 5% minor allele frequency [51] on the samples with at least 50-fold viral read coverage. Only the iSNV sites with a coverage of at least 50 reads were retained. All the identified variants were annotated using SnpEff (version 5.1) with default settings. All the plots were visualized using the R package ggplot (v.3.3.0) [52]. Among the major vANI95 clusters, samples with more than 50-fold viral read coverage were subjected to coinfection detection. The genomic positions of the iSNVs from these samples were compared against those of the cSNVs from other individuals in the same vANI95 cluster. Samples were considered to be coinfected if more than 3 iSNVs shared the same genomic positions as the cSNVs from one of the other individuals.

### Mantel test of geographic associations

A consensus nucleotide sequence for each individual was generated using the SNVs detected above. The genetic distance between pairwise sequences was calculated using the dist.dna function of the R package ape (version 5.5) [53]. The geographic spherical distance (km) was calculated using the sampling locations in the R package geosphere (version 1.5–18) (https://github.com/rspatial/geosphere). The Mantel test was performed using the mantel and mantel.partial functions of the R package vegan (version 2.6–4) [54]. For virome comparisons, the dissimilarities among viral communities (viral prevalence within geographic sites) and among sample viromes (viral abundance within samples) were measured with the Bray‒Curtis distance and Jaccard distance, respectively, using the R package proxy (version 0.4‒27). The Bray‒Curtis distances of the overall viral community at the genus and family levels for each bat taxon were used to generate dendrograms using the hclust function and subsequently compared to the host phylogeny.

## Results

### Sampling information

**Fig. 1:**
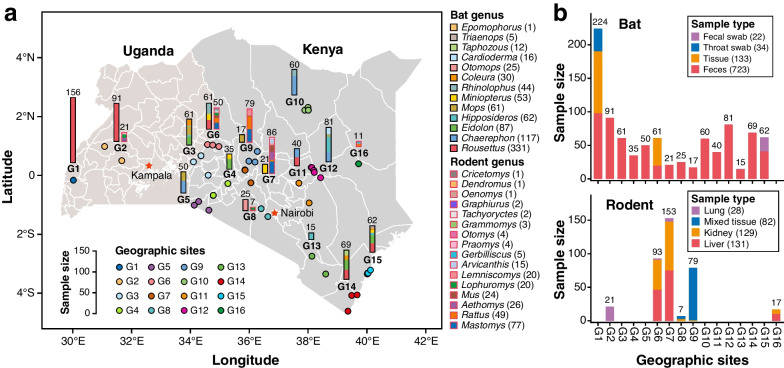
Sample information and geographic background. **a **Distribution of sampling locations and host species of bat and rodent samples. The height of each histogram represents the number of samples with distinct host individuals. Histograms representing bat and rodent samples are indicated with black edges and red edges, respectively. The color within the histogram represents the host genus according to the legend. The color of the circles represents the geographic site. For clarity, the number of individuals per host genus is summarised for each geographic site. The 49 sampling locations were clustered into 16 geographic sites with a maximum within-site spherical distance of less than 90 km. **b **Distribution of bat and rodent samples by sample type and geographic site. The color represents the sample type

A total of 1282 samples (912 samples from 844 bats and 370 samples from 250 rats) were collected from 49 sampling locations across Kenya and Uganda in East Africa between 2014 and 2019 (Additional file 1: Table S1). To compare viral communities across geographical areas, we grouped the 49 sampling locations into 16 geographic sites (G1–G16) according to the spherical distance of one location from the other (Fig. 1a; see the “Methods” section). The samples included 24 bat (13 genera, eight families) and 22 rodent (16 genera, four families) species (Additional file 2: Fig. S1), which was confirmed by morphological identification and comparison of the assembled contigs against public mitochondrial sequences with at least 95% nucleotide identity. The bat samples included a total of 745 fecal samples (including fecal swabs), 133 tissue samples, and 34 throat swab samples (Fig. 1b). The rodent samples included lung, kidney, and liver tissues (as described in Additional file 1: Table S1).

### Large-scale metatranscriptomes revealed substantial undescribed viral diversity

Metatranscriptomic sequencing of the 1282 samples generated 38.3 Tb of raw data, with a mean data size of 29.9 Gb per sample (Additional file 1: Table S1). After filtering low-quality reads, rRNA and duplicated reads further yielded 8.6 Gb (mean) of rRNA-free clean data per sample. Subsequent de novo assembly and viral genome annotation (Additional file 2: Fig. S2) of the 1282 metatranscriptomes revealed at least 8500 viral sequences associated with vertebrates, insects, plants, and fungi. Only the viral sequences most similar to the known viruses with vertebrate hosts were defined as vertebrate-associated viruses and further characterized (see the “Methods” section).

**Fig. 2:**
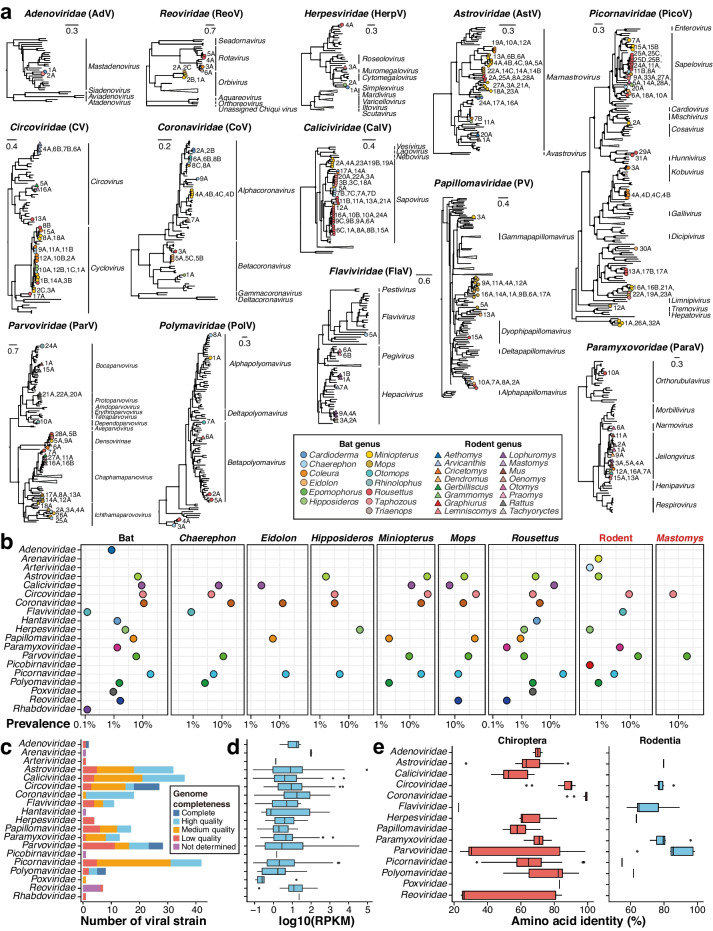
Diversity of vertebrate-associated viruses in East Africa. **a** Maximum likelihood phylogenetic trees of major viral families harboring replication-associated proteins (Additional file 1: Table S4). The name of the viral family is shown above each tree. The solid black circles on each branch node represent bootstrap values above 50. The tip nodes on each tree represent an average of 95% nucleotide identity (vANI95) representatives identified in the present study, with the color indicating the host genus and the shape representing the host order, as listed in the bottom panel. **b** Prevalence among distinct host individuals of each viral family in bats, rodents, and major (*n* > 50) host genera. **c** Completeness of representative genomes in each viral family, with the color indicating genome completeness. **d** Distribution of viral abundance (measured by reads per kilobase of transcript per million reads mapped (RPKM)) in each viral family. **e** Amino acid identity of replication-associated proteins (RAP-AAI) between the viruses identified in this study and the viruses collected from public databases in bat and rodent samples

The 251 identified vertebrate-associated virus ANI95 clusters (hereafter vANI95) comprised 164 RNA vANI95 clusters (133 at the vANI80 level) and 87 DNA vANI95 clusters (75 at the vANI80 level) from 19 viral families (Additional file 1: Table S2). Among them, 212 vANI95 clusters were identified in bats, and 39 vANI95 clusters were identified in rodents (Fig. 2a). For bat viruses (*n* = 212), the major viral families included *Adenoviridae* (*n* = 2), *Astroviridae* (*n* = 31), *Caliciviridae* (*n* = 36), *Circoviridae* (*n* = 21), *Coronaviridae* (*n* = 18), *Herpesviridae* (*n* = 3), *Papillomaviridae* (*n* = 17), *Paramyxoviridae* (*n* = 6), *Parvoviridae* (*n* = 19), *Picornaviridae* (*n* = 41), *Polyomaviridae* (*n* = 7), and *Reoviridae* (*n* = 7). For rodent viruses (*n* = 39), the major viral families included *Circoviridae* (*n* = 6), *Flaviviridae* (*n* = 10), *Paramyxoviridae* (*n* = 7), and *Parvoviridae* (*n* = 9). Viruses of *Picornaviridae*, *Coronaviridae*, and *Circoviridae* were most prevalent in bats, whereas *Parvoviridae*, *Circoviridae*, and *Flaviviridae* were most prevalent in rodents (Fig. 2b). However, the dominant viral families in bats and rodents may also indicate tissue tropism due to the major difference between their sample types.

All the vANI95 representative sequences (*n* = 251) were de novo assembled from metatranscriptomic data, which included 88 medium-quality (completeness > 50%) and 102 complete/high-quality (completeness > 90%) genomes (Fig. 2c). Nonetheless, the quality of identified genomes may be underestimated due to the discovery of highly divergent viral genomes and the underrepresentation of African bat and rodent-borne viruses in existing databases, as well as the insensitivity of CheckV to segmented viruses [34]. Among the families with multiple vANI95 clusters (*n* > 1), *Adenoviridae*, *Coronaviridae*, and *Parvoviridae* exhibited relatively high viral abundance (Fig. 2d; Additional file 1: Table S3). In particular, most (17/18) vANI95 representatives of *Coronaviridae* were high-quality genomes, suggesting that a high viral abundance may contribute to genome assembly quality (Fig. 2c, d).

Using RAPs as evolutionary markers (Additional file 1: Table S4), only 13% of the vANI95 representatives were closely related to known viruses (> 90% average amino acid identity, RAP-AAI) (Additional file 1: Table S2), reflecting that most of the viral diversity in East Africa remains to be described. The novelty of these 251 vANI95 representatives varied across viral groups. Among the major viral families with more than five vANI95 representatives, the median RAP-AAI against the known viruses was relatively high for *Coronaviridae* (99.51%) and *Circoviridae* (88.82%) (Fig. 2e). In contrast, the median RAP-AAI values of the remaining major viral families were less than 80%. In addition to the limited surveillance of viruses with zoonotic potential in Africa, the dependence on targeted screening rather than metagenomic/metatranscriptomic approaches has led to imbalanced efforts across viral groups in previous surveillance. Seven viral families (*Astroviridae*, *Caliciviridae*, *Paramyxoviridae*, *Pirconaviridae*, *Circoviridae*, *Papillomaviridae*, and *Parvoviridae*) composed phylogenetic clades with at least five potential novel vANI95 clusters. Notably, a group of 36 vANI95 clusters of *Sapovirus* was newly identified in *Caliciviridae*, tripling the size of known bat viral genomes in this genus (Fig. 2a). Other examples of expanded viral genera included *Mamastrovirus* (*n* = 32, *Astroviridae*), *Jeilongvirus* (*n* = 13, *Paramyxoviridae*), *Sapelovirus* (*n* = 20, *Pirconaviridae*), *Cyclovirus* (*n* = 21, *Circoviridae*), and *Chaphamaparvovirus* (*n* = 10, *Parvoviridae*).

**Fig. 3:**
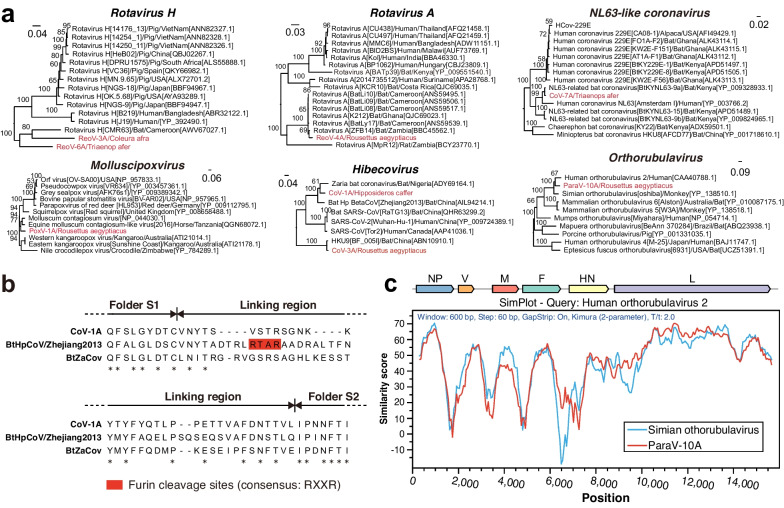
Evolution of viruses related to human and domestic animal pathogens. **a** Maximum likelihood phylogenetic trees of viruses harboring replication-associated proteins. All the trees are midpoint rooted, with the scale bar representing the count of amino acid substitutions per site. The name of the viral group is shown above each phylogeny. For each tree, bootstrap values are marked on the branch node. The virus names included the viral species, strain name, host species, and sampling country. The viruses identified in the present study are indicated in red. **b** Sequence alignment of the spike protein in hibecoviruses, with the furin cleavage site highlighted in red boxes. **c** Genomic similarity among related orthorubulaviruses with human orthorubulavirus 2 (parainfluenza 2) as a query

Several vANI95 clusters were evolutionarily related to viruses that infect humans and/or domestic animals (> 70% RAP-AAI) (Fig. 3a). In *Coronaviridae*, we identified an NL63-like vANI95 cluster (CoV-7A from *Triaenops* bats) in which the S gene nested within the 299E-like group, in contrast to other bat NL63-like viruses [55] (Additional file 2: Fig. S3). Given that the S-gene of HCoV-NL63 is likely derived from a 229E-like virus circulating in *Hipposideros* bats [55], our findings revealed another NL63-like virus harboring the 229E-like S-gene. In addition, we identified one vANI95 cluster of *Hibecovirus* (CoV-1A) from *Hipposideros* bats with high similarity to the Hp-betacoronavirus/Zhejiang2013 (Zhejiang2013) in China (87.8% RAP-AAI) [56]. *Hibecovirus* is a subgenus of *Betacoronavirus* that encompasses several notable human pathogens, including SARS-CoV-2. In contrast to that of Zhejiang2013, the spike proteins of both CoV-1A and BtZaCoV did not contain a furin cleavage motif (RXXR) (Fig. 3b), supporting an independent origin of the furin cleavage site within Zhejiang2013 after its split from the other two *Hibecovirus* relatives in Africa. In *Paramyxoviridae*, one vANI95 cluster of orthorubulavirus (ParaV-10A) from *Rousettus aegyptiacus* was closely related to human parainfluenza virus 2 (HPIV-2) (78.8% RAP-AAI) (Fig. 3a, c). This orthorubulavirus vANI95 cluster represents the first reported wildlife orthorubulavirus genome related to this human respiratory disease-causing agent. In *Poxviridae*, one vANI95 cluster (PoxV-1A) from *R*. *aegyptiacus* was closely related to the human molluscum contagiosum virus (MCV) (genus: *Molluscipoxvirus*) (83.5% RAP-AAI) (Fig. 3a), a pathogen causing chronic skin lesions in humans [57]. Interestingly, several genes associated with PoxV-1A matched those of eukaryotic proteins (Additional file 1: Table S5). These genes included a gene similar to the SPRY domain-containing SOCS box protein (SPSB) found in the lesser hedgehog tenrec (*Echinops telfairi*), which is involved in the regulation of nitric oxide (NO) levels. Nitric oxide plays a defensive role against infections by promoting the proteasomal degradation of inducible nitric oxide synthase (iNOS) [58]. Therefore, host-derived genes may play roles in regulating host antiviral immunity.

### The evolutionary conservation of replication-associated proteins varies across viral families

**Fig. 4:**
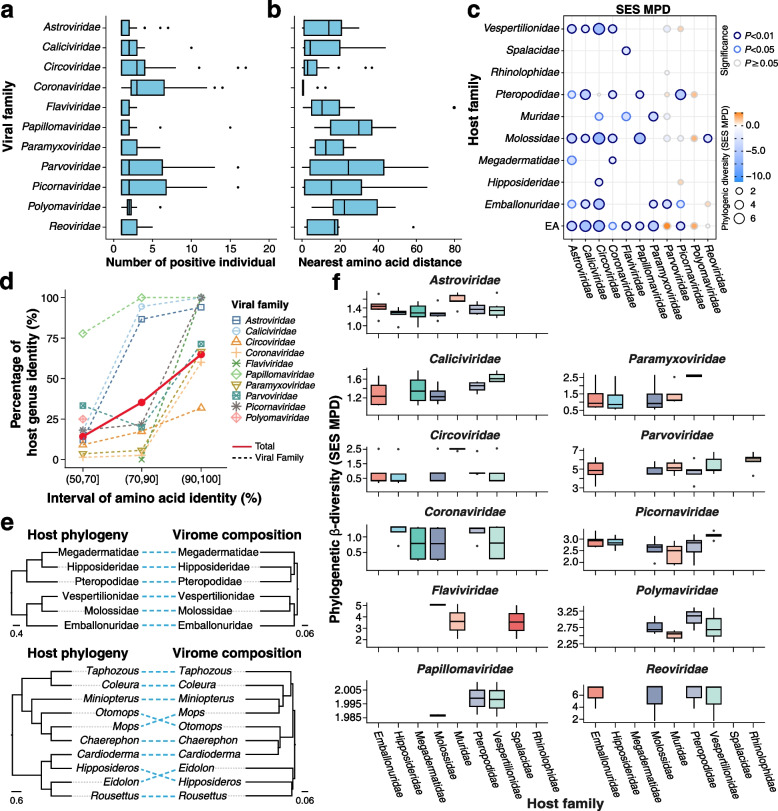
Phylogenetic diversity of viruses among host groups. **a** Distribution of vANI95 cluster sizes in each viral family. The cluster size represents the number of distinct host individuals in each viral ANI95 cluster. **b** Nearest amino acid distance of each representative vANI95 among the viral families. **c** Phylogenetic diversity as estimated by the standardized effect size of the mean phylogenetic distance (SES MPD) across host families that have at least three viral records. **d** Host sharing and RAP-AAI between viral pairs of the identified viruses. **e** Cophylogeny of host taxonomy and virome composition at the family and genus levels. A dendrogram of the virome composition was generated from the Bray‒Curtis distance among viral communities clustered at 65% RAP amino acid identity. **f** Phylogenetic *β* diversity as estimated by the SES-MPDs among host families

The richness and diversity of closely related viruses may reflect the potential for genetic interactions and the phenotypic plasticity of a viral group. We estimated the viral population size of each vANI95 cluster by using the number of individuals infected with that viral cluster. The cluster size of vANI95 varied across viral families (Fig. 4a). For example, *Circoviridae* and *Coronaviridae* had the largest median cluster sizes, while *Astroviridae* had a relatively small cluster size although more vANI95 representatives were identified within this family.

RAPs play a major role in genetic exchange among closely related viruses [59]. We compared the sequence conservation among RAPs of vANI95 representatives using the nearest amino acid distance (Fig. 4b). A smaller amino acid distance indicates a higher level of sequence conservation through evolutionary processes. In our comparison across viral families, *Coronaviridae* exhibited the smallest average nearest distance, implying greater conservation, followed by *Caliciviridae* and *Circoviridae*. On the other hand, *Astroviridae*, *Parvoviridae*, and *Picornaviridae* displayed the largest average distances, suggesting less conservation of RAPs. The results were similar when we compared the within-genus phylogenetic diversity among viral families using the MPD (Additional file 2: Fig. S4a) and the MNTD of RAPs (Additional file 2: Fig. S4b), reflecting varied RAP diversity among viral families. Interestingly, the nearest amino acid distance of vANI95s marginally correlated with their population size (Additional file 2: Fig. S4c), suggesting that a conserved RAP might be vital for maintaining the viral population size.

To quantify host specificity across viral families, we compared the observed MPD and MNTD against a random distribution using the SES of the MPD and MNTD (Fig. 4c, Additional file 2: Fig. S4d). A negative SES-MPD indicates phylogenetic clustering. In contrast, a negative SES-MNTD indicates a phylogenetic position near the tip. We found significant and negative SES-MPD and SES-MNTD values in most available viral and host families, suggesting a high level of host specificity and phylogenetic structuring of viruses in East Africa. Nonetheless, *Parvoviridae*, *Polyomaviridae*, and *Picornaviridae* had the most positive (but nonsignificant) SES-MPDs among the host families, suggesting a weak effect of host structuring in those viral families.

We further quantified host specificity along evolutionary distance using the proportion of vANI95 pairs sharing at least one host genus within each RAP-AAI interval (Fig. 4d, Additional file 1: Table S6). The viral families showed host genus specificity only when the RAP-AAI reached 65%. When the viral RAPs were clustered at 65% AAI, the structuring of the Bray‒Curtis distance among viral communities was largely concordant with the host–taxonomic relationship at both the family and genus levels in bats (Fig. 4e), suggesting the major role of host evolution in structuring the viral community among host taxa. Notably, *Astroviridae, Caliciviridae*, *Papillomaviridae*, and *Picornaviridae* exhibited the highest host specificity, with at least 60% of the hosts sharing viral pairs when the RAP-AAI was greater than 90%. In contrast, *Coronaviridae* and *Circoviridae* had the lowest host specificity. To further quantify host turnover among viral families, we assessed the phylogenetic variation of viral communities across host taxa. Viral families showed varied structuring of phylogenetic similarity and positive SES-MPDs among host families (Fig. 4f). Nonetheless, *Coronaviridae* and *Circoviridae* had the lowest SES-MPD values among the host families, suggesting greater phylogenetic turnover in these two viral families.

### Viral genomic comparison revealed abundant signals of recombination and reoccurring mutations related to viral sharing

**Fig. 5:**
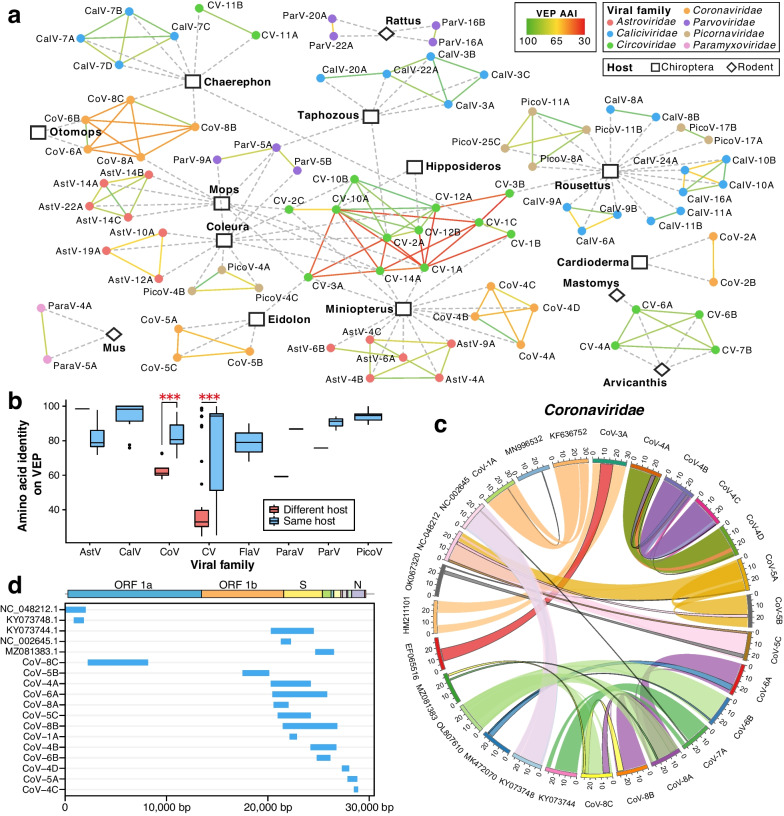
Recombination and host specificity. **a** Network of host range and genome variability among vANI95 clusters. Edges represent viral entry proteins (VEPs) dissimilarity, and nodes represent hosts and viruses. Only the vANI95 clusters with closely related neighbors (RAP-AAI > 90%) were included in this network. **b** Divergence of VEPs among closely related viruses in East Africa. **c** Recombination within *Coronaviridae*. Genomes involved in recombination are represented as segments surrounding the circles. The major parent, minor parent, and recombinant were connected according to the recombination position of the recombinant. The colors of the ribbons are consistent with those of the major/minor parent of the recombinant. The major parents are connected with ribbons with black borders. Minor parents are connected with ribbons without borders. **d** Genomic distribution of recombination breakpoints in *Coronaviridae*. For each recombinant (as an individual gray line), genomic regions flanked by recombination breakpoints are indicated as blue rectangles

The vastly expanded genomic resources comprise abundant evolutionary signals of antigen-related regions. We therefore explored the genomic variability among closely related vANI95 clusters (RAP-AAI > 90%), with a particular focus on VEPs (Additional file 1: Table S4). The most closely related vANI95 pairs (RAP-AAI > 90%) exhibited similar VEPs (VEP-AAI > 70%) (Fig. 5a, Additional file 1: Table S7)*.* In contrast, the closely related vANI95 pairs of *Coronaviridae* and *Circoviridae* had highly divergent VEPs (VEP-AAI < 70%) (Fig. 5a), indicating their greater capacity to maintain a viral population with diverse VEPs potentially facilitating host adaptation. This difference was more evident when their host genera were different (Fig. 5b), suggesting that VEPs could be informative when retracing recent viral sharing.

Despite most vANI95 clusters exhibiting strong host specificity, 27 vANI95 clusters or close relatives (RAP-AAI > 90%) were detected in multiple host genera (Additional file 2: Fig. S5), involving viruses of the *Coronaviridae*, *Circoviridae*, and *Parvoviridae families* and *Astroviridae*. Interestingly, a group of *Circoviridae* vANI95 clusters with similar RAPs were detected among each other in the *Hipposideros*, *Rousettus*, *Coleura*, *Mops*, and *Miniopterus* bats*.* It seems that *Coleura* or *Mops* bats were the hubs for bat host‒virus associations in *Circoviridae*. The *Circoviridae* vANI95 clusters also showed high similarity to the cycloviruses identified in rodents and shrews (Additional file 2: Fig. S6) [60], suggesting potential transmission between bats and small terrestrial mammals. Despite similar RAPs, these cyclovirus-like vANI95s exhibited high VEP (capsid) divergence (VEP-AAI < 70%). The cyclovirus-like vANI95s that shared similar VEPs (VEP-AAI > 70%) were all detected in *Hipposideros and Taphozous*. In comparison, the VEP-AAI among the others detected in *Mops* and *Miniopterus* ranged from 29.2 to 48.4% (Fig. 5a). The RAPs and capsid proteins of the genus *Cyclovirus* also showed substantial phylogenetic incongruence, suggesting frequent recombination within this genus (Additional file 2: Fig. S6). To infer recent viral evolution within each vANI95, we also compared the viral genomes using between-host cSNVs. However, we did not observe enrichment of cSNVs in the VEP regions of *Circoviridae* viruses (Additional file 1: Table S8), suggesting that the divergence of capsid proteins was driven by recombination rather than rapid mutation.

The coronaviruses identified in our study exhibited abundant recombination signals among the high-quality or complete vANI95 representative genomes and publicly available genomes (Fig. 5c, Additional file 1: Table S9). The breakpoints of recombination were located mainly near the S-gene or the 3’ end of the genome (Fig. 5d), revealing a similar recombination hotspot as that reported recently for SARS-CoV-2 [61]. This difference might be an innate feature of coronaviruses related to the long-range genetic interactions associated with the secondary structure of the coronavirus genome [62].

Among the *Coronaviridae* vANI95 clusters with recombination signals, we observed recombination among five closely related vANI95 clusters of alphacoronaviruses from three free-tailed bat (Molossidae) genera (*Otomops*, *Mops*, *Chaerephon*) (Additional file 2: Fig. S7a, b). Despite the high similarity in most genomic regions, the S1 regions of the *Otomops* CoVs (CoV-6A and CoV-6B) and the *Mops* CoV (CoV-8A) were highly divergent and more closely related to Chaerephon CoV-WA3607 and Chaerephon CoV-CpYN11, respectively.

Using CoV-8A as an example, we further identified repeated SNPs at the same genomic positions after the split of three CoV-8 vANI95s, suggesting recurrent mutations during long-term evolution (Additional file 2: Fig. S8a). CoV-8A and other *Coronaviridae* vANI95s also showed enrichment of cSNVs in the VEP regions (Additional file 1: Table S8), reflecting a faster evolutionary rate within the antigen-related regions. In contrast, cSNVs were infrequently found within the RAP regions of coronaviruses (Additional file 2: Fig. S8b), which is consistent with the high degree of conservation of RAPs across the vANI95 clusters. It is likely that RAPs are subject to strong stabilizing selection, preserving their sequence similarity across viruses, while VEPs are more prone to diversifying selection, which encourages variation. These differential mutation patterns between RAPs and VEPs underscore the distinct evolutionary pressures acting on these regions.

### Cocirculation and occasional gene flow contribute to extensive viral diversity

**Fig. 6:**
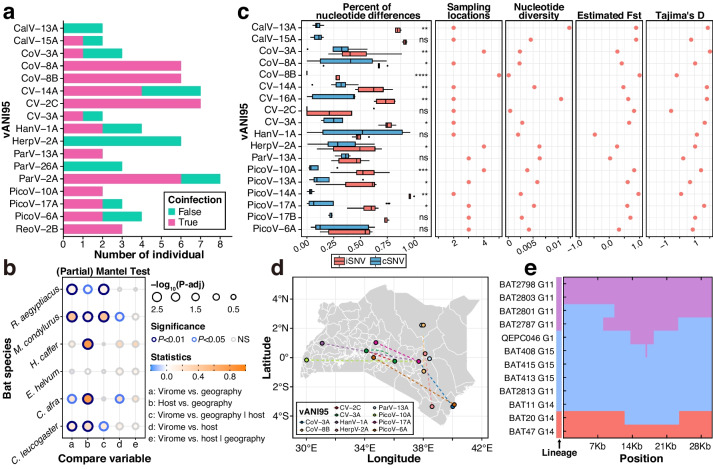
Repeat mutations, cocirculation, and occasional gene flow. **a** Number of coinfected individuals within major vANI95 clusters. **b** Mantel tests and partial Mantel tests comparing host genetic distance, geographic distance, and structure of virome dissimilarity with *P* values adjusted by the Benjamini and Hochberg methods. **c** Population genetic statistics (Pi, Fst, Tajima’s D, and number of geographic sites) of major vANI95 clusters across the sampling sites. **d** Geographic connection (> 200 km) of most similar viral pairs within the same vANI95 cluster. **e** Impact of recombination on the genome-wide population structure of CoV-3A

Geographic distribution is a key factor when structuring viral populations. A comparison of cSNVs revealed varied nucleotide diversity among vANI95 populations (with at least four members), ranging from 0.011 (CoV-8B) to 1.23% (CalV-13A) (Additional file 1: Table S10). The iSNV data indicated that virus-positive samples frequently carried genetically distinct viruses of the same vANI95 cluster (Fig. 6a). Interestingly, our data further revealed a positive correlation between the nucleotide diversity of vANI95 clusters and their coinfection rate (Additional file 2: Fig. S9, Additional file 1: Table S11), suggesting that viral population diversity may have a broad impact on driving intrahost viral genomic interactions in wildlife.

Both genetic distance and virome similarity indicated major dispersal limitations across geography (Fig. 6b). Most major bat species showed that the Jaccard distance among sample viromes was correlated with geographic distance (Fig. 6b). Notably, we observed the weakest geographic associations with host mitochondrial genetic distance in *R.* *aegyptiacus* and *Eidolon* *helvum*, suggesting frequent migration in fruit bats (Fig. 6b). Two-thirds (12/18) of the vANI95 clusters across geography showed significant nucleotide differences between sampling locations (Fig. 6c). Nonetheless, we observed 41 vANI95 clusters spanning more than 200 km (Additional file 1: Table S2), including 8 vANI95 clusters with nearly identical (nucleotide identity > 99.95%) viral pairs from bats across large geographic distances (> 200 km) in *Coronaviridae* (CoV-3A, 8B), *Circoviridae* (CV-2C, 3A), and *Picornaviridae* (PicoV-6A, 10A, 13A, and 10A), suggesting recent gene flow across geography (Fig. 6d). Interestingly, these potential recent transmission routes seemed to be located around central Kenya, suggesting that this region might be the centre of viral transmission in East Africa (Fig. 6d).

To demonstrate the impact of geographic transmission on viral genomic diversity, we compared the consensus viral genomes across geographic locations using CoV-3A from *R.* *aegyptiacus* as an example (Fig. 6e). The CoV-3A virus had a high RAP-AAI (> 99%) relative to that of HKU9, which was previously found to be a marked genomic polymorphism [63]. Clustering of the consensus CoV-3A genomes revealed that two out of the three lineages were detected only in G11 and G14. Nonetheless, the remaining lineage of CoV-3A occurred at four geographic sites (G1, G11, G14, and G15), spanning 1100 km. Recombinants between coexistent lineages of the same site were also detected in G11 and G14, suggesting that infrequent gene flow maintained the genetic diversity of local regions despite major dispersal limitations. The results also demonstrated the potential use of deep sequencing for tracing genomic interactions and targeting viral hot spots for viral surveillance in wildlife.

## Discussion

Using metatranscriptomic sequencing, we characterized and compared the viromes of 1282 bat and rodent samples from Kenya and Uganda. The genomic and geographic profiling data of the 251 viruses identified reveal the broad diversity and complexity of the viral communities in East Africa, indicating the need for in-depth viral surveillance in this area.

One surprising aspect was the dominant proportion (218/251) of potential novel vANI95 clusters, reflecting the geographic specificity of both viral and host distributions in East Africa. Given the evolutionary continuity of vertebrate and invertebrate viromes [14], many viruses related to the ones identified here remain to be discovered. Another reason for the substantial number of potential novel viruses is the imbalance of research efforts among viral taxa, as indicated by the vast difference in the proportion of potential novel viruses among viral families and host taxa. In our study, the assembled viral genomes tremendously increased surveillance sensitivity in East Africa, accelerating the discovery of viruses with spillover risk within the previously neglected viral groups. The structuring of viral communities among bat genera confirmed their broad coevolution with host taxa. Nonetheless, we identified several viruses related to recent virus sharing, suggesting potential cryptic circulation among wildlife species.

Among the surveyed wildlife taxa, it is hypothesized that fruit bats harbor virulent zoonotic pathogens, including Marburg virus [64], henipavirus [65], and Sosuga virus [66]. In our study, viruses phylogenetically related to human pathogens were observed, strengthening the importance of surveillance in *R. aegyptiacus *bats. For example, our data showed that a vANI95 representative, ParaV-10A, was closely related to HPIV-2 and simian parainfluenza virus. HPIV-2 and 4 are the causal agents of respiratory disease and can lead to severe outcomes [67]. Considering the recent identification of a close relative of parainfluenza virus 4 (another virus of human health concern, *Orthorubulavirus*) in *Eptesicus* bats [68], bats may harbor the common ancestor of diverse parainfluenza viruses circulating in humans. In *R. aegyptiacus*, our study revealed a molluscum contagiosum-like poxvirus (PoxV-1A) related to the human MCV, which can persist for weeks or even years instead of causing acute disease [69–71]. In humans, MCV encodes genes involved in anti-inflammatory activities via the inhibition of nuclear factor-κB (NF-κB) [70], a key transcription factor involved in the regulation of iNOS when activated by cytokines and infectious organisms. Interestingly, our study showed that the PoxV-1A genome encodes a homolog of the SPSB from vertebrates. In vertebrates, SPSBs regulate oxidative stress by mediating the proteasomal degradation of iNOS. The expression of the viral homolog of SPSBs in PoxV-1A may allow it to counteract innate antiviral immunity, ensuring long-term within-host replication of the virus. In recent years, wildlife-human contact has become more common due to habitat encroachment and increased consumption of wildlife in rural areas [1]. The constant monitoring of zoonoses at the human-livestock-wildlife interface should be prioritized.

Among other hosts, we identified viruses potentially involved in recent viral sharing or host-sharing events. In particular, our study revealed that bat cycloviruses are closely related to cycloviruses in shrews, suggesting the possibility of cross-species transmission between bats and shrews or rodents [60]. Another example here is the recombination-related viral sharing of coronaviruses among three bat genera in the *Molossidae* family. Interestingly, viruses of the *Coronaviridae* and *Circoviridae* families have the highest VEP divergence while maintaining relatively high RAP conservation, indicating that viruses of those families generally possess a more diverse genetic pool with which a virus could interact. Among closely related viruses, viruses of the *Coronaviridae* and *Circoviridae* genera also exhibited larger population sizes, frequent recombination, and viral sharing. Indeed, viruses with higher RAP conservation are expected to be more likely to exchange genetic material if recombination occurs only between viruses with sufficient genome similarity. A population with frequent recombination is expected to be more effective at accumulating adaptive variants and more tolerant to variant fixation caused by genetic drift, as most such mutations are deleterious [7]. The enrichment of recombination breakpoints surrounding the S gene region of coronaviruses further suggested that genomic regions with greater variability may be subject to more effective selection. Here, we hypothesized that there are at least two viral evolution modes involved. Viruses with rapidly evolving RAPs tend to have smaller populations and limited recombination capacity among diverse genetic pools, leading to rapid speciation. Viruses with more conserved RAPs tend to occur in larger populations, allowing higher genomic diversity and more frequent recombination. The ability to effectively recombine within a diverse genetic pool can give these viruses an advantage in terms of virus sharing. Given the contrasting conservation patterns of RAPs and VEPs among viral families, genomic comparisons should be more informative when tracing viral sharing among related viruses in future research.

A previous study demonstrated limited dispersal and substantial genetic drift of viromes in wildlife [12], which are expected to cause genetic structuring across geography. Despite major dispersal limitations, our study showed that coinfection of distinct viral lineages is frequent in bats. Consequently, the transmission of a few viral lineages causes a detectable effect on the local genomic composition through recombination. Our study demonstrated the feasibility of tracing viral transmission hotspots by detecting genomic interactions. However, given the relatively small sample size of the viral population in our study, a more systematic comparison among viral groups requires a larger scale of viral surveillance in future research.

## Conclusions

In summary, the identified vertebrate-associated viruses exhibit vast viral diversity, with up to 87% of potential novel viruses being associated with recombination, geographic isolation, and host adaptation in East Africa. In particular, the *Circoviridae* and *Coronaviridae* viral families frequently recombine, causing genetic changes associated with virus–host interactions and host shifts among host genera. Despite major dispersal limitations, recurrent mutations, cocirculation, and occasional gene flow contribute to high viral diversity. With the landscape of vertebrate-associated viral communities described here, our study demonstrates the broad application of metatranscriptomic sequencing in revealing the genomic associations underlying the diversity and dispersal of viral communities in East Africa. This study also provides a feasible approach for retracing genomic interactions and targeting viral hot spots for effective pathogen surveillance in the future.

### Supplementary Information

**Additional file 1.**

**Additional file 2.**

**Additional file 3.**

**Additional file 4.**

**Additional file 5.**

## Data Availability

Clean read data have been deposited into the SRA database (Bioproject accession PRJNA1083756) and China National GeneBank DataBase, CNGBdb (project accession: CNP0003101). The viral genome sequences have been deposited in the NCBI GenBank database (Bioproject accession PRJNA1083756) and the CNGB Sequence Archive (CNSA, https://db.cngb.org/cnsa/) of the CNGBdb (project accession: CNP0004728). Viral genomic sequences, intermediate data, and code related to this work are available on GitHub at https://github.com/alexzrren/EastAfrica_Virome.
