# Supplementary material for: Substantial viral diversity in bats and rodents from East Africa: insights into evolution, recombination, and cocirculation
Source: Microbiome. 2024 Apr 10;12:72. doi: 10.1186/s40168-024-01782-4 (PMC11005217; doi:10.1186/s40168-024-01782-4)
Supplement: Supplementary file 3 — Additional file 2. [file 40168_2024_1782_MOESM2_ESM.docx]

## Additional File 2

#####
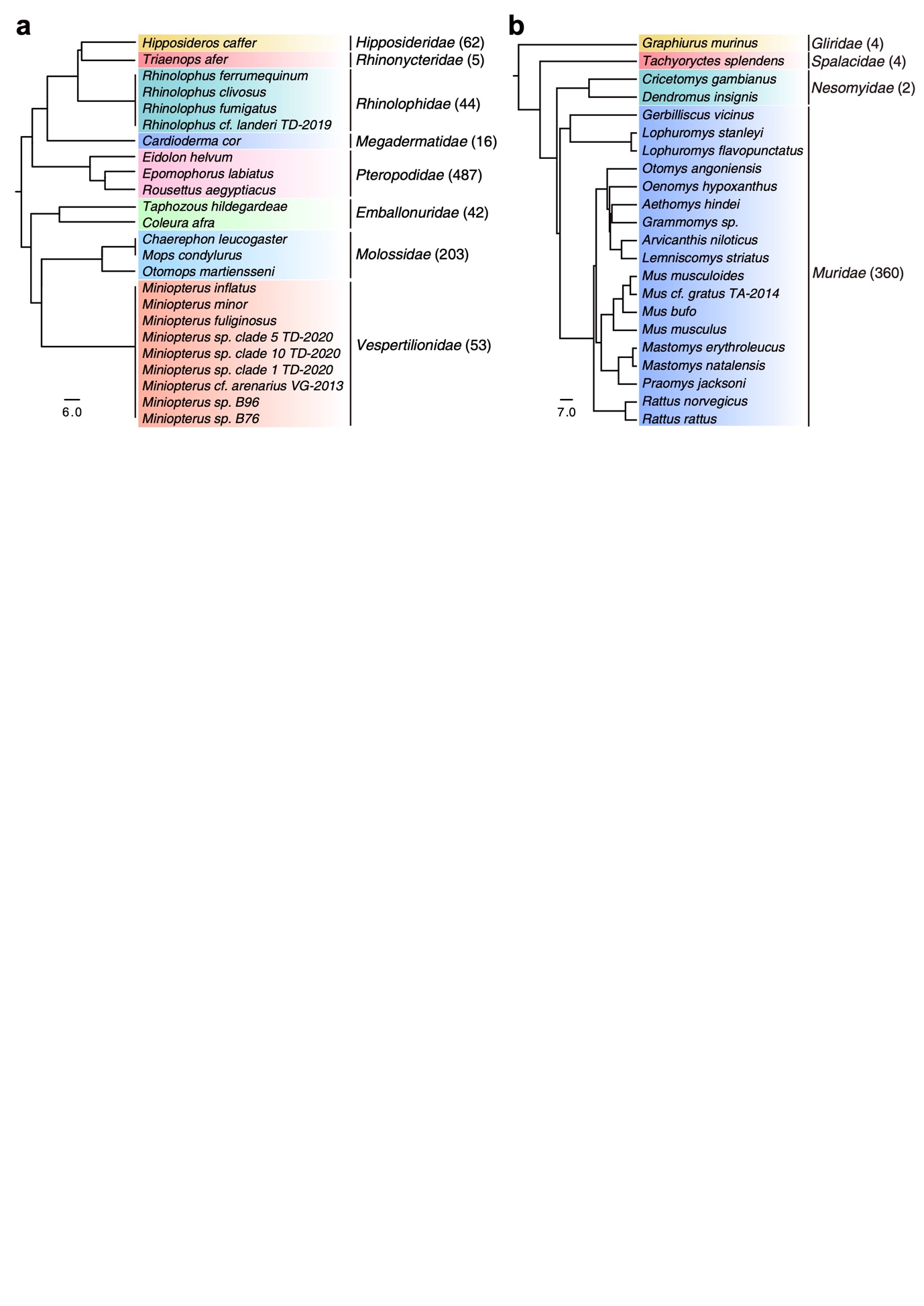
Fig. S1. Taxonomy of surveyed host species

**a** Phylogeny of the bat species surveyed in this study. **b** Phylogeny of the rodent species surveyed in this study. **Host phylogenies of host species were derived from public subsets of the mammalian phylogeny** (http://vertlife.org/phylosubsets).

##### Fig. S2. Workflow for virus sequence identification

**The reads were filtered to remove ribosomal RNA (rRNA), adapter, low-quality, duplicate and low complexity sequences**. **The filtered reads were *de novo* assembled and compared against viral proteins from the nonredundant (NR) protein database and the RefSeq and IMG-VR databases. The matched contigs were annotated using the Contig Annotation Tool (CAT) and compared against the nucleotide (NT) database and reference genomes of *Chiroptera* and *Rodentia* to remove host sequences. The proteins translated from each viral contig were compared against representative replication-associated proteins derived from the NR database. For RNA viruses, only the viruses matching the RdRp conserved domains from the conserved domain database (CDD) were considered further. To annotate vertebrate-associated viruses, all viral sequences were compared against proteins in the Virus‒Host Database (VHDB). Only the viral sequences that were most similar to a vertebrate-associated virus were selected for further analyses. The completeness of each viral sequence was assessed, after which the sequences were clustered at 80% nucleotide identity (vANI80) and 95% (vANI95). Within each clustering level, the viral sequence with the longest length was selected to represent the viral species/strain.**


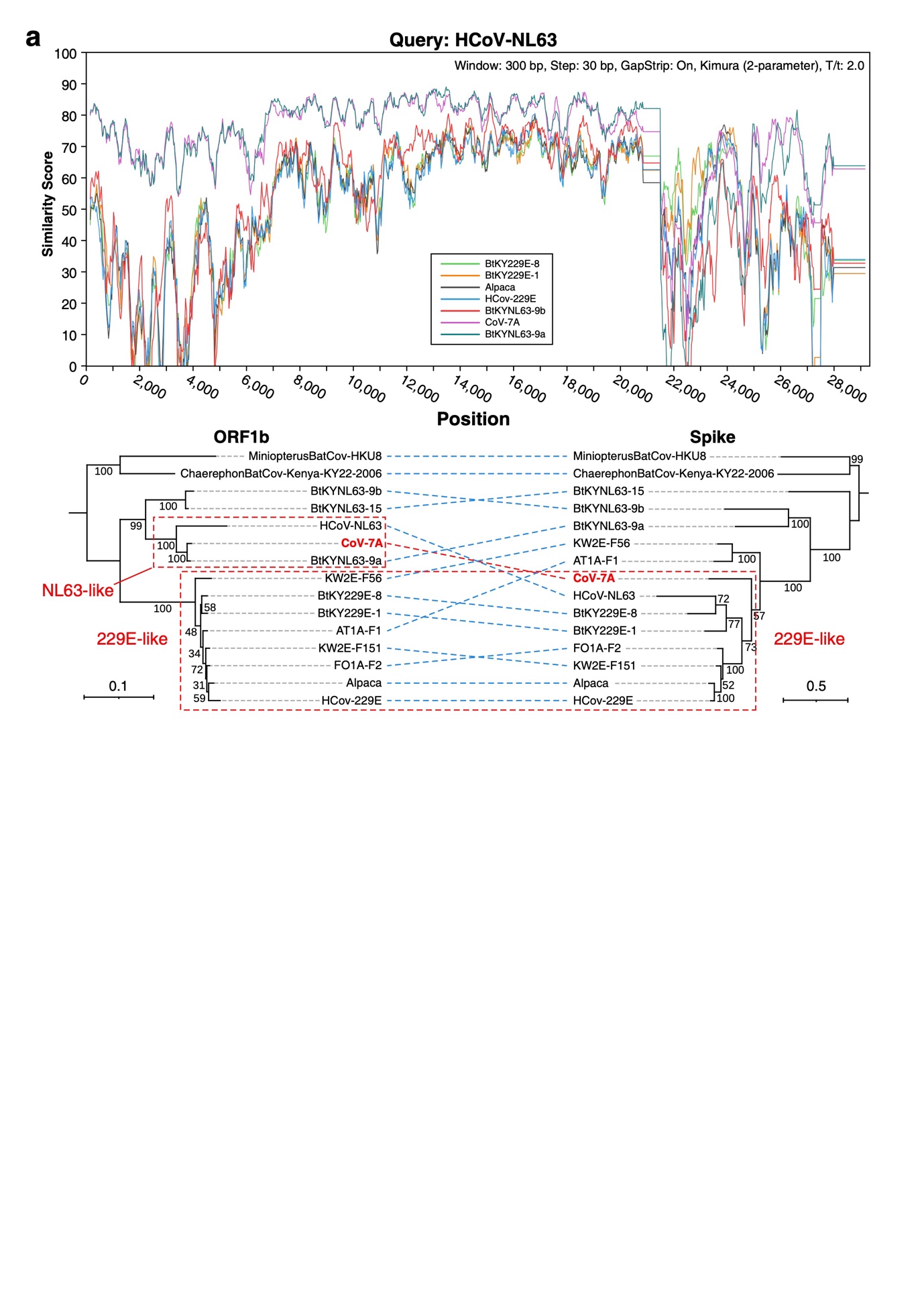


##### Fig. S3. Evolution of CoV-7A

Maximum likelihood phylogenetic trees of open reading frame (ORF)1b (left) and the spike region (right) of CoV-7A-related viruses. The trees are midpoint rooted, with the scale bar representing the count of amino substitutions per site.


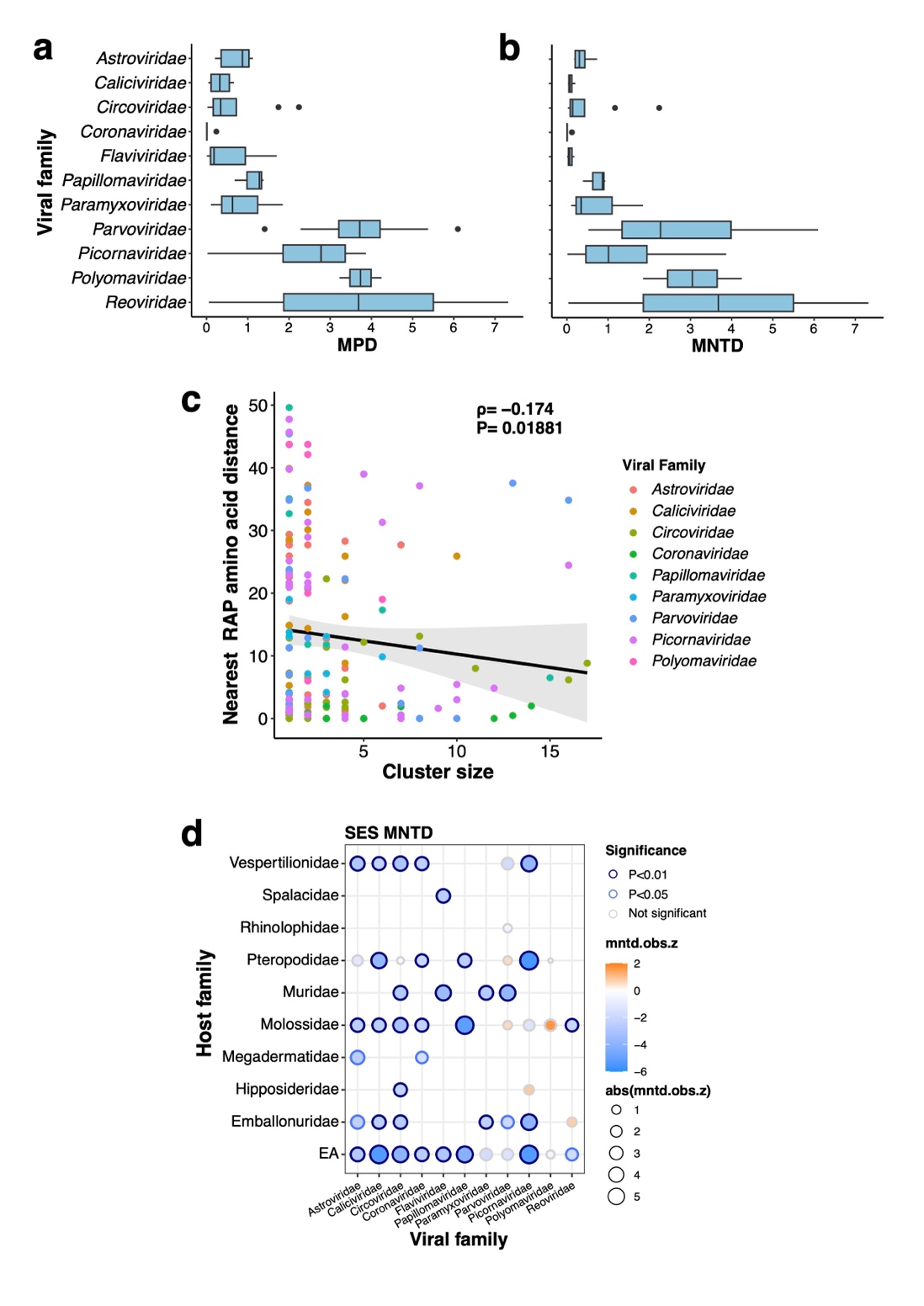


##### Fig. S4. Phylogenetic diversity of viruses among host groups

**a** Mean pairwise distance (MPD) among vANI95s within each viral family. **b** Mean nearest-pairwise distance (MNTD) among vANI95s within each viral family. **c** Spearman correlation between the nearest RAP amino acid distance to each vANI95 and the cluster size. **d** Phylogenetic diversity of the host families with at least three viral records as estimated by the standardised effect size of mean nearest taxon distance (SES-MNTD).


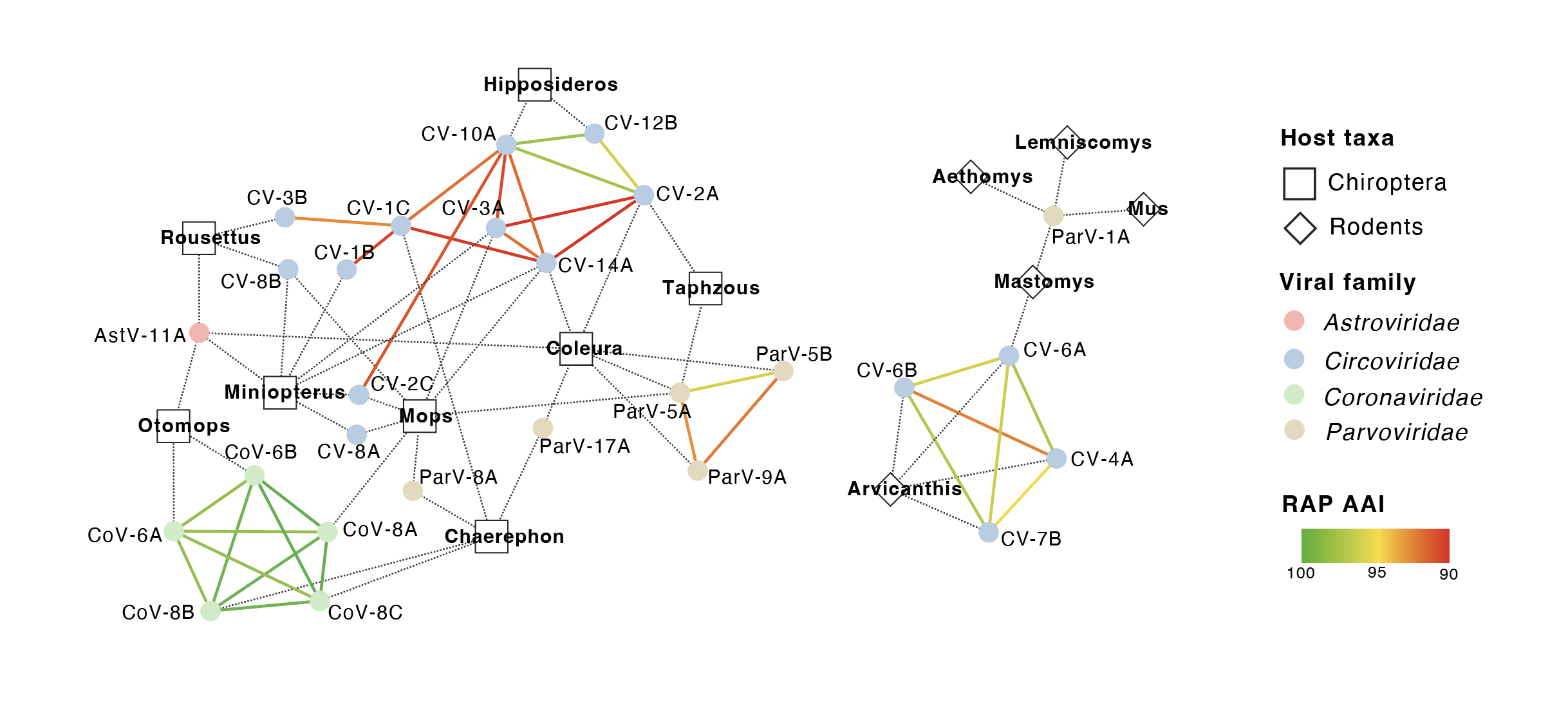


##### Fig. S5. Network of closely related vANI95 clusters associated with different host genera

Edges represent the RAP similarity. The nodes represent the host and virus.

##### Fig. S6. Phylogenies of capsid and replication-associated proteins in *Cyclovirus*

Phylogenetic tree of capsid protein (left) and replication-associated protein (right) with bootstrap support values labelled on each branch node, using chicken-associated cyclovirus 2 as the outgroup. The viruses identified here are shown in red.

##### Fig. S7. Recombination analysis of CoV-6 and CoV-8

**a** Sliding window analysis of the genomic similarity between CoV-6A and CoV-8A and their closely related coronaviruses. The putative recombination breakpoints are indicated by dashed lines. The similarities against different reference sequences are indicated by different colours. **b** Maximum likelihood phylogenetic trees of the ORF1b (left) and S1 regions (right) of the coronaviruses identified in the bat genera *Chaerephon*, *Otomops* and *Mops* and their close relatives, with BtYN63-9a serving as the outgroup. The trees are midpoint rooted, with the scale bar representing the count of amino substitutions per site.


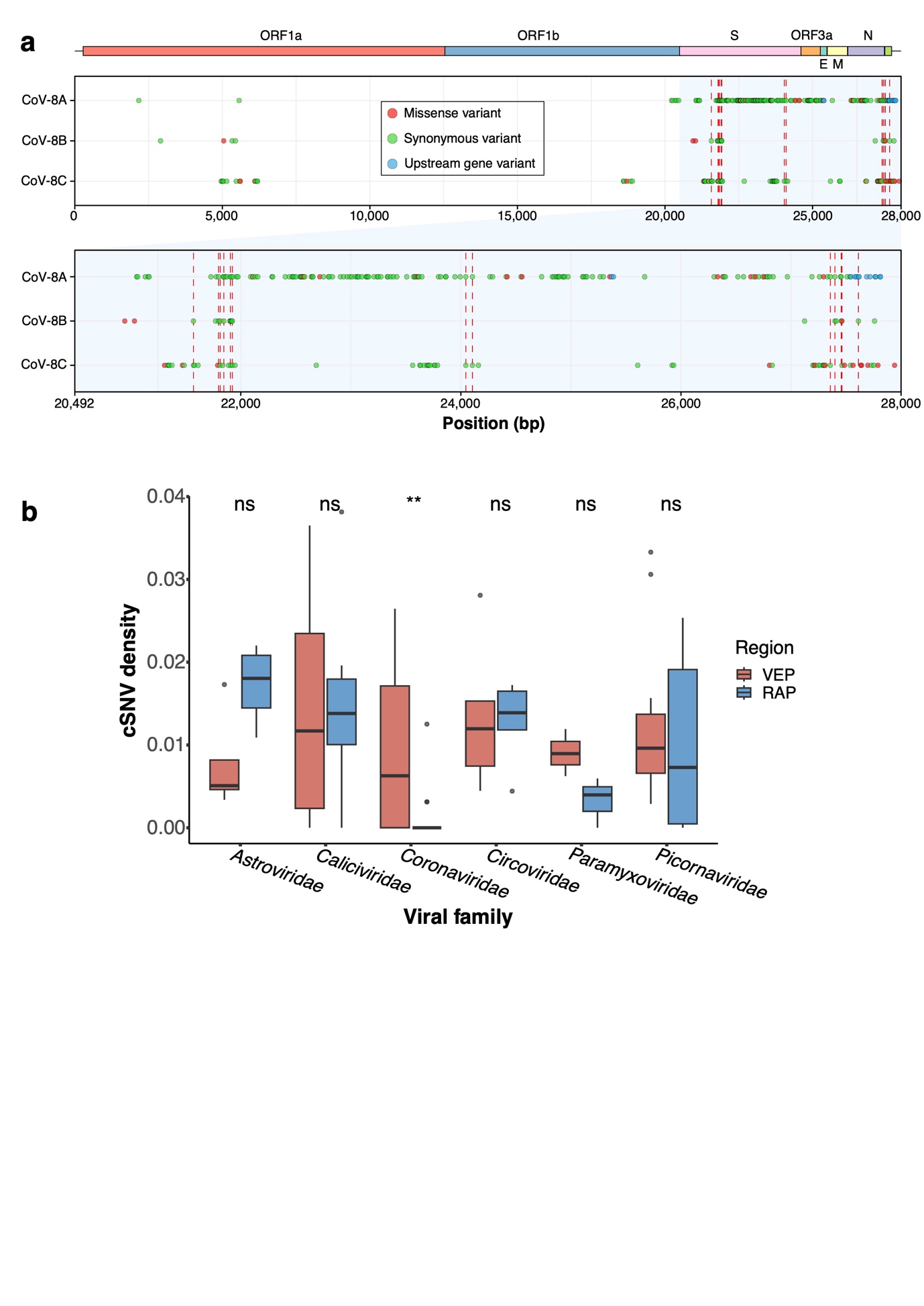


##### Fig. S8. Polymorphic sites in the genomes of *Coronaviridae*

**a** Variable sites across CoV-8 genomes. Polymorphic sites are indicated in circles along the CoV-8 genome. The colours represent the biological effects of the mutations. Recurrent mutations among at least two of the three vANI95 clusters are indicated by red dashed lines. **b** The cSNV density of the vANI95 cluster with at least 10 cSNV sites within the VEP and RAP regions.


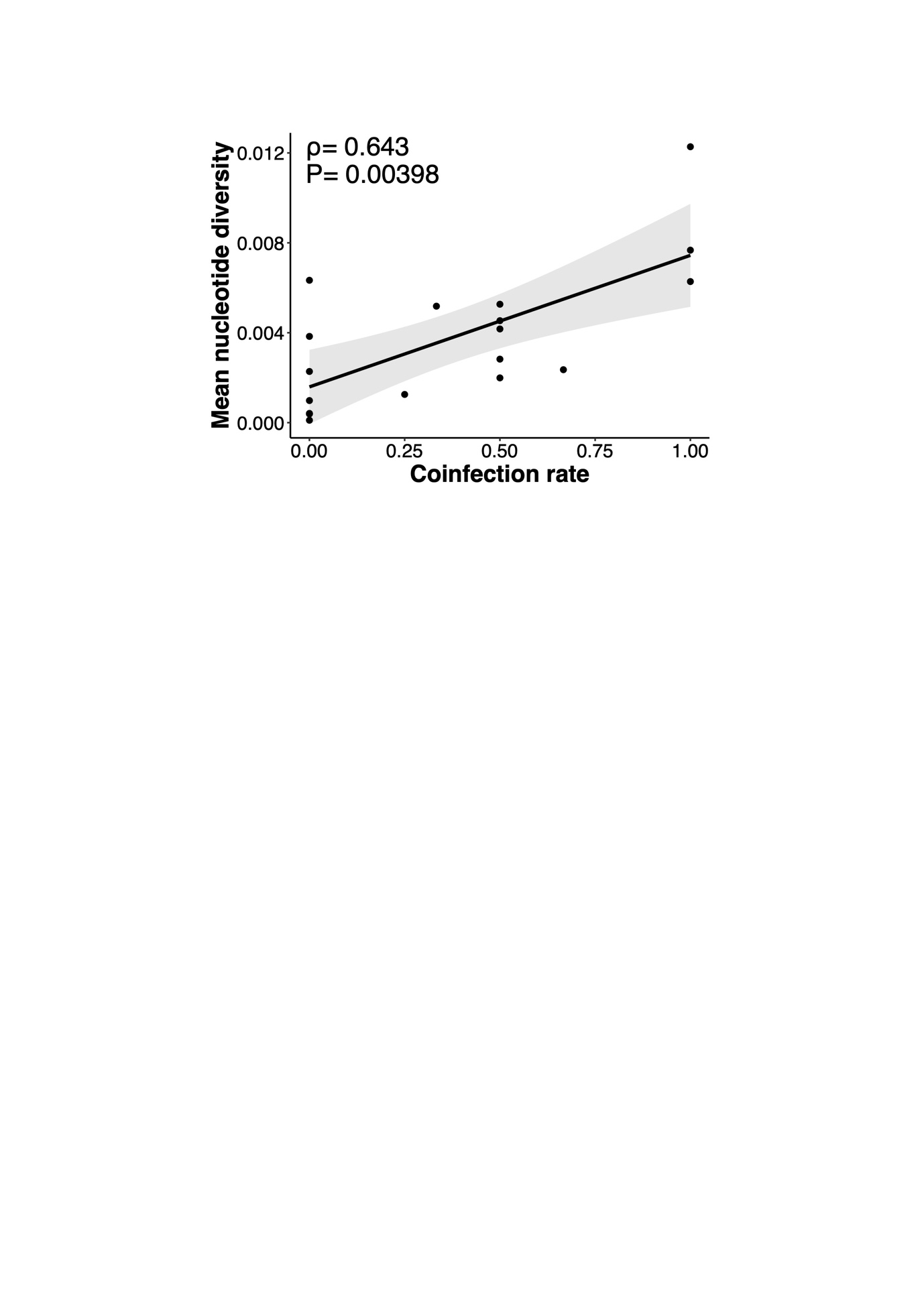


##### Fig. S9. The correlation between nucleotide diversity and the coinfection rate in major vANI95 clusters.
